# Differential expression of alarmins—S100A9, IL-33, HMGB1 and HIF-1α in supraspinatus tendinopathy before and after treatment

**DOI:** 10.1136/bmjsem-2017-000225

**Published:** 2017-05-31

**Authors:** Michael J Mosca, Andrew J Carr, Sarah J B Snelling, Kim Wheway, Bridget Watkins, Stephanie G Dakin

**Affiliations:** Nuffield Department of Orthopaedics, Rheumatology and Musculoskeletal Sciences, Botnar Research Centre, Oxford, UK

**Keywords:** Alarmins, Tendinopathy, Tendon, Inflammation, S100A9, HMGB1, IL-33, HIF-1

## Abstract

**Background:**

Alarmins, endogenous molecules released on tissue damage have been shown to play an important role in inflammatory musculoskeletal conditions including fracture repair andrheumatoid arthritis. However, the contribution of alarmins to the pathogenesis of tendon disease is not fully understood.

**Methods:**

We investigated expression of alarmin proteins (S100A9, high-mobility group box 1 (HMGB1) and interleukin-33 (IL-33) and hypoxia-inducible factor 1α (HIF-1α), a subunit of an oxygen sensitive transcription factor, in three cohorts of human supraspinatus tissues: healthy (n=6), painful diseased (n=13) and post-treatment pain-free tendon samples (n=5). Tissue samples were collected during shoulder stabilisation surgery (healthy) or by biopsy needle (diseased/treated). Immunohistochemistry was used to investigate the protein expression of these factors in these healthy, diseased and treated tendons. Kruskal-Wallis with pairwise post hoc Mann-Whitney U tests were used to test for differences in immunopositive staining between these tissue cohorts. Additionally, costaining was performed to identify the cell types expressing HIF-1α, S100A9, IL-33 and HMGB1 in diseased tendons.

**Results:**

Immunostaining showed HIF-1α and S100A9 were increased in diseased compared with healthy and post-treatment pain-free tendons (p<0.05). IL-33 was reduced in diseased compared with healthy tendons (p=0.0006). HMGB1 was increased in post-treatment pain-free compared with healthy and diseased tendons (p<0.01). Costaining of diseased tendon samples revealed that HIF-1α, S100A9 and IL-33 were expressed by CD68+ and CD68− cells, whereas HMGB1 was predominantly expressed by CD68− cells.

**Conclusions:**

This study provides insight into the pathways contributing to the progressionand resolution of tendon disease. We found potential pro-inflammatory and pathogenic roles for HIF-1α and S100A9, a protective role fornuclear IL-33 and a potentially reparative function for HMGB1 in diseased supraspinatus tendons.

**Key points**

- We found differential protein expression of alarmins in healthy and diseased human supraspinatus tendons, before and after treatment.
- The cell types expressing S100A9, interleukin-33 (IL-33) and hypoxia-inducible factor 1α (HIF-1α) included macrophages and tendon stromal cells.
- S100A9 and HIF-1α may have pro-inflammatory effects in tendon disease, nuclear IL-33 may be protective against pro-inflammatory stimuli and high-mobility group box 1 may have a potential role in tendon healing.
- Improving understanding of the role of alarmins in tendon disease will facilitate identification of potential therapeutic targets for patients with tendinopathy.

## Background

Tendon disease represents a significant clinical challenge and is associated with substantial disability among ageing and athletic populations.^1^ Supraspinatus tendinopathy is one of the most common orthopaedic conditions presenting to clinicians with an estimated prevalence of 4%–26%.^2^ The importance of inflammation in the pathogenesis of tendon disease is a subject of ongoing debate. Over the past two decades, tendon disease has been characterised as a ‘degenerative condition’.^3^ More recently, the identification of key immune cell populations and signalling molecules as important regulators in the initiation and propagation of other musculoskeletal diseases, including rheumatoid arthritis (RA) and spondyloarthropathy, has led to a re-emergence of interest in the role of inflammation in tendon disease.^4–7^

The term ‘tendinopathy’ has been popularised to reinforce the notion that the underlying disease mechanisms are not fully understood.^8^ However, there is growing support for the contribution of immune cells and inflammatory mediators to the development of tendinopathy.^9–13^ Recent research showed that numbers of macrophages and mast cells are significantly increased in tendinopathic compared with healthy tendon tissues, and that inflammatory and fibrotic cytokines, peptides and growth factors have altered expression profiles in diseased compared with healthy tendons.^14 15^ We recently reported that inflammatory signatures changed throughout different stages of severity in tissue samples from patients with supraspinatus tendinopathy.^13^ However, the mechanisms by which inflammation is perceived by resident tendon cells remains poorly understood.

Alarmins, endogenous molecules released on tissue damage, are key effectors in the activation of the immune system that may be important in the pathogenesis of tendon disease.^16^ Alarmins are structurally diverse host proteins with intracellular or intra nuclear functions, which act in both host-defence and tissue repair.^17^ These molecules ‘alarm’ the immune system by upregulating signalling pathways including nuclear factor-κβ (NF-κβ), interferons (IFNs) and cyclooxygenases, which have downstream effects on tendon repair processes.^17^ Studies of alarmins in RA have implicated these molecules in the failure of acute inflammatory resolution, resulting in dysregulated processes and development of chronic inflammation.^18^ There is a paucity of research on alarmins in tendon disease.^19 20^ To our knowledge, no study has previously investigated alarmin protein expression in non-ruptured diseased supraspinatus tendons before and after treatment using tendon matched healthy control tissues. Moreover, there are little data regarding the potential roles of S100A9 and HMGB1 in tendinopathy.

The purpose of this study was to investigate expression of three alarmins in human supraspinatus tendons that have been implicated in sustaining chronic inflammation in diseased musculoskeletal tissues: 1) S100A9 2) high-mobility group box 1 (HMGB1) and 3) interleukin-33 (IL-33).^18^ Although there is a growing list of alarmins in the literature, these are the most thoroughly studied and implicated in other musculoskeletal diseases yet have not been investigated in tendinopathy. Additionally, we investigated expression of hypoxia-inducible factor-1α (HIF-1α), a subunit of HIF-1, an oxygen-dependent heterodimeric transcription factor implicated in pro-inflammatory and profibrotic diseases.^21 22^ HIF-1 has been implicated as a critical regulator of early tendinopathy but has not yet been investigated in non-ruptured, diseased tendons.^20^ We investigated protein expression of these mediators in three cohorts of human supraspinatus tissues: 1) healthy ‘control’ tendons, 2) painful diseased tendons (not torn) and 3) tendons from patients who became asymptomatic after subacromial decompression (SAD) treatment. An additional objective was to identify the cell types expressing alarmins in diseased tendon tissues. We hypothesised that expression of S100A9, HMGB, IL-33 and HIF-1α would be increased in painful diseased compared with healthy tendons. We further hypothesised that macrophages and resident tendon cells in diseased tissues would express these proteins.

## Methods

### Overview

Ethical approval for this study was granted by the local research ethics committee, Oxfordshire REC B references 10/H0402/24, 09/H0605/111 and South Central Oxford B reference 14/SC/0222. Full informed consent according to the Declaration of Helsinki was obtained from all patients. Tissue was obtained from three well-phenotyped patient groups: 1) 6 healthy supraspinatus tendons from patients undergoing shoulder stabilisation, 2) 13 diseased supraspinatus tendons (intact and not torn) from patients with shoulder pain undergoing SAD and 3) 5 pain-free post-treatment supraspinatus tendons from patients who previously had SAD surgery for shoulder impingement (tissue collected 1–3 years post-SAD). Samples were collected by a consultant orthopaedic surgeon during surgery (healthy and diseased) or under local anaesthetic (treated group). All patients included in the study completed the Oxford Shoulder Score (OSS), a validated pain and functional outcome measure prior to tissue collection and/or surgery.^23^ All tissue samples were processed, embedded, sectioned and stained using immunohistochemistry (IHC) to quantify protein expression of S100A9, HMGB1, IL-33 and HIF-1α. Statistical analysis and sample size justification were determined from previous studies that were sufficiently powered using similar IHC protocols.^11 13^ Fluorescent immunostaining was also performed on diseased supraspinatus tissues to identify whether macrophages (CD68+ cells) previously identified in diseased tendons^13^ also expressed HIF-1α, S100A9, IL-33 and HMGB1.

### Patient cohort and tissue collection

All patients were recruited from orthopaedic referral clinics where the structural integrity of the supraspinatus tendon was determined ultrasonographically. Samples of healthy supraspinatus tendon tissues were collected from patients recruited to the Nuffield Orthopaedic Centre (NOC) from orthopaedic referral clinics for shoulder instability (table 1). During the surgical procedure a tendon biopsy was collected. Diseased supraspinatus tendon tissues were collected from patients presenting to orthopaedic referral clinics for shoulder pain. Patients presenting to the shoulder clinic had failed non-operative treatments and had experienced pain for a minimum of 6 months. Tendon tissues were collected via ultrasound-guided biopsy prior to surgical subacromial decompression. The biopsy was taken using a Tru-Cut needle 5 to 10 mm posterior to the anterior edge of the supraspinatus tendon. This validated biopsy technique is described in detail elsewhere.^24^ Post-treatment pain-free supraspinatus tendon tissues were collected from patients who underwent the referral process for chronic shoulder pain/supraspinatus tendinopathy, had a SAD procedure and who were clinically asymptomatic for at least 1 year after surgery. Patients in the pain-free group had significant pain before SAD that resolved post-treatment, evidenced by a post-treatment median OSS of 48 (range, 45–48). Pain-free post-treatment samples were collected 1–3 years after treatment using a percutaneous ultrasound-guided biopsy technique under local anaesthetic. Exclusion criteria for any patient included significant problems in the other shoulder, significant neck problems, RA, systemic inflammatory disease, osteoarthritis, previous shoulder surgery and dual shoulder pathological lesions.

**Table 1:** Patient cohort details for tendon tissue samples used in the current study

Tissue group	n	Median age (years)	History	Prior treatment	Median OSS
Healthy	6	24 (range: 19–26)	Shoulder instability	N/A	33 (range: 11–44)
Diseased	13	45 (range: 36–62)	Painful tendinopathy	N/A	30 (range: 16–36)
Pain-free post-treatment	5	57 (range: 43–72)	Painful tendinopathy	SAD	48 (range: 45–48)

N/A, not available; OSS, Oxford Shoulder Score, SAD, subacromial decompression.

### Tissue processing

Samples of tendons were immersed in 10% buffered formalin for 0.5 mm/hour. After formalin fixation, tendon samples were processed in a Leica ASP300S tissue processor and subsequently embedded in paraffin wax; 4 µm tissue sections were cut using a RM2135 microtome (Leica Microsystems) and baked onto adhesive glass slides at 60°C for 30 min and 37°C for 60 min.

### Immunohistochemistry

Prior to antibody staining and antigen retrieval, slides were baked at 60°C for 60 min. Deparaffinisation and antigen retrieval was performed using an automated PT link (Dako, heat-mediated antigen retrieval at high pH). An Autostainer Link 48 (Dako) was used to perform antibody staining using an EnVision FLEX visualisation system. Details of antibodies and their working dilutions are shown in supplementary table S1. Using protocols provided by the manufacturer, antibody binding was visualised using a FLEX 3,3′-diaminobenzidine (DAB) substrate working solution and haematoxylin counterstain (Dako). After staining, slides were taken through graded industrial methylated spirit and xylene and mounted in Pertex mounting medium (Histolab). Isotype control staining was performed to confirm specificity of staining, whereby the primary antibody was substituted for universal isotype control antibodies. The murine universal isotype control used was a cocktail of mouse IgG1, IgG2a, IgG2b, IgG3 and IgM (Dako IR750). The universal isotype control for rabbits was an immunoglobulin fraction of serum from non-immunised rabbits, solid-phase absorbed (Dako IR600).

10.1136/bmjsem-2017-000225.supp1 Supplementary table 1

### Image acquisition and quantitative analysis for IHC

All images were acquired on an inverted microscope using Axiovision software (Zeiss). Twenty images of each stained section were acquired in a systematic manner at ×100 magnification with oil immersion by a single-blinded investigator. If the tissue section was not large enough to capture 20 images, images were taken until the available tissue was exhausted. ImageJ (National Institutes of Health, Bethesda, Maryland, USA) was used to analyse the acquired images. Previously validated algorithms that quantify DAB staining by a colour deconvolution methods were used in analysis.^25 26^ For each antibody, the colour deconvolution threshold was manually adjusted to best represent immunopositive staining. Depending on expression pattern (cytoplasmic/nuclear or extracellular), specific algorithms were used to quantify immunostaining. For antibodies that showed nuclear and/or cytoplasmic staining, a cell ratio was calculated: positively stained cells/total cells (S100A9, HMGB1 and IL-33). Once positive cells and total cells were counted for each image, the results were summed for each sample to give total positive cells/total cells. For antibodies that showed staining of the extracellular matrix, a ratio of the stained area was calculated: stained area ratio = total positively stained pixels in the image/total pixels of the image (HIF-1α). Once positive pixels and total pixels were quantified for each image, the results were summed to give the stained area ratio: total number of stained pixels/total pixels of all images. For each sample, immunopositive staining was normalised to the number of haematoxylin-counterstained nuclei within the field of view to account for cellularity of the tissue sample.

### Immunofluorescence

Staining protocols are adapted from a previously validated protocol.^13^ Prior to antibody staining and antigen retrieval, slides were baked at 60°C for 60 min. Slides were taken through deparaffinisation and target retrieval steps (high pH heat-mediated antigen retrieval) using an automated PT Link FLEX TRS system (Dako). Slides were placed in a humid chamber throughout staining to prevent drying.

Prior to antibody staining, tissues were blocked in 5% normal goat serum (Sigma G9023) in phosphate buffered saline (PBS) for 45 min at room temperature to prevent non-specific antibody binding. Slides were then incubated with the selected primary antibody combination and diluted in 5% normal goat serum in PBS at room temperature for 2 hours (see online supplementary table S1). Isotype controls were completed to confirm staining seen in tendon tissues was specific; the primary antibody was substituted for universal isotype control antibodies available from the manufacturer (Dako). The universal isotype control for mice was a cocktail of mouse IgG1, IgG2a, IgG2b, IgG3 and IgM (Dako IR750). The universal isotype control for rabbits was an immunoglobulin fraction of serum from non-immunised rabbits, solid-phase absorbed (Dako IR600).

After primary antibody staining, slides were washed three times in phosphate buffered saline with Tween 20 (PBST) solution for 5 min each time. Slides were then incubated with three secondary antibodies: 1) goat antimouse IgG1 (Southern Biotech), 2) Alexa Fluor 568 goat antimouse IgG2a and 3) Alexa Fluor 633 antirabbit IgG (Life Technologies). Secondary antibodies were incubated at 1:200 dilution in PBS containing 5% normal equine serum (Sigma) for 2 hours at room temperature. After secondary antibody staining, slides were washed three times in PBST. POPO-1 nuclear counterstain (Life Technologies) was then applied to all slides diluted in PBS containing 5% Saponin (Sigma) for 20 min at room temperature. Slides were then washed with PBST and incubated in a solution of 0.1% Sudan Black B (Applichem A1407) in 70% ethanol for 5 min to quench tissue autofluorescence. After staining was complete, slides were mounted in VectaShield fluorescent mounting medium (H1000) and a coverslip secured. After mounting, slides were stored in a humid box at 4°C until imaging.

### Immunofluorescence image acquisition and analysis

Immunofluorescence images were acquired on a Zeiss LSM 710 confocal microscope using a ×40 oil immersion objective following a previously described protocol.^13^ Two-dimensional reconstructions were created using ZEN 2009 (Zeiss).

### Statistical analyses

Statistical analyses were performed using GraphPad Prism V.7 (GraphPad Software). Normality was tested using Shaprio-Wilk test for normality. Kruskal-Wallis with pairwise post hoc Mann-Whitney U tests were used to test for differences in immunopositive staining between healthy, diseased and post-treatment pain-free sample groups. These tests were performed on DAB-stained immunohistochemical images. Statistical significance was set at p<0.05.

## Results

### HIF-1α and S100A9 proteins are increased in diseased supraspinatus tendons

**Figure 1:**
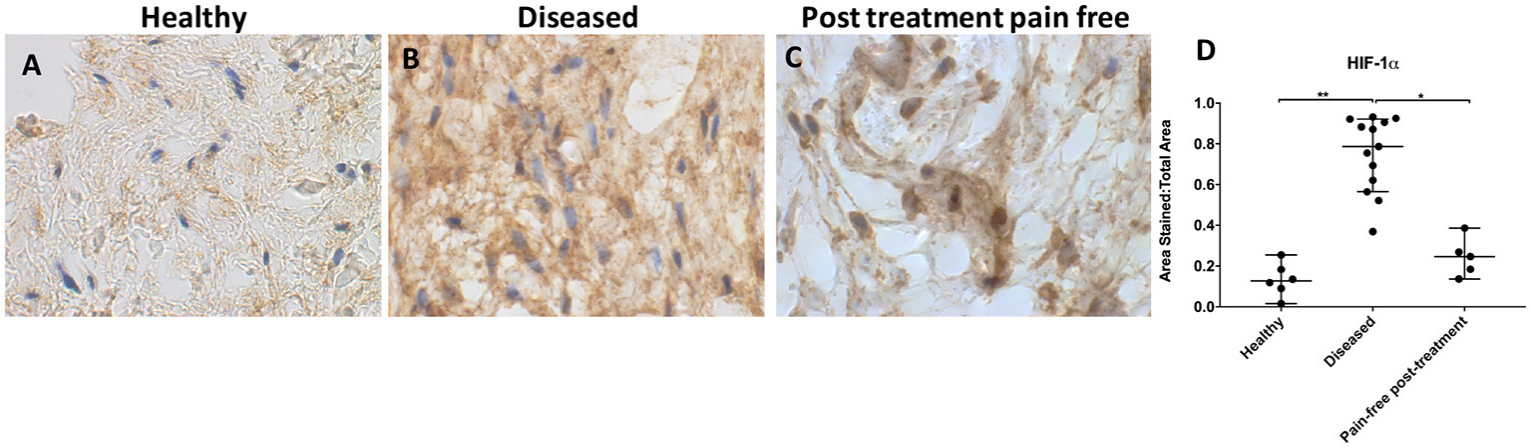
Representative images of 3,3′-diaminobenzidine immunostaining (brown) for hypoxia-inducible factor 1α (HIF-1α) in (A) healthy, (B) diseased and (C) postsubacromial decompression pain-free human supraspinatus tendons. Nuclear counterstain is haematoxylin (blue). Scale bar=20 µm. (D) Quantitative analysis of immunopositive staining for HIF-1α. Bars represent median value with 95% CIs. Data were analysed using Kruskal-Wallis test with pairwise post hoc Mann-Whitney U test; *p<0.05, **p<0.01, ***p<0.001.

HIF-1α staining was increased in diseased compared with healthy supraspinatus tendons (p=0.0002, 6.2-fold increase; figure 1A, B and D). Healthy tendons showed low-level extracellular HIF-1α immunostaining. HIF-1α staining was reduced in post-SAD treatment patients whose pain resolved compared with diseased tissue from symptomatic patients (p=0.03, 3.2-fold decrease (figure 1C)). There was no significant difference in HIF-1α staining between healthy and treated tissue (p=0.97). Diseased tendons showed high-level HIF-1α staining throughout the samples, although nuclear staining was not observed. Pain-free treated tissues showed moderate HIF-1α staining that was also strongly present in the nucleus.

**Figure 2:**
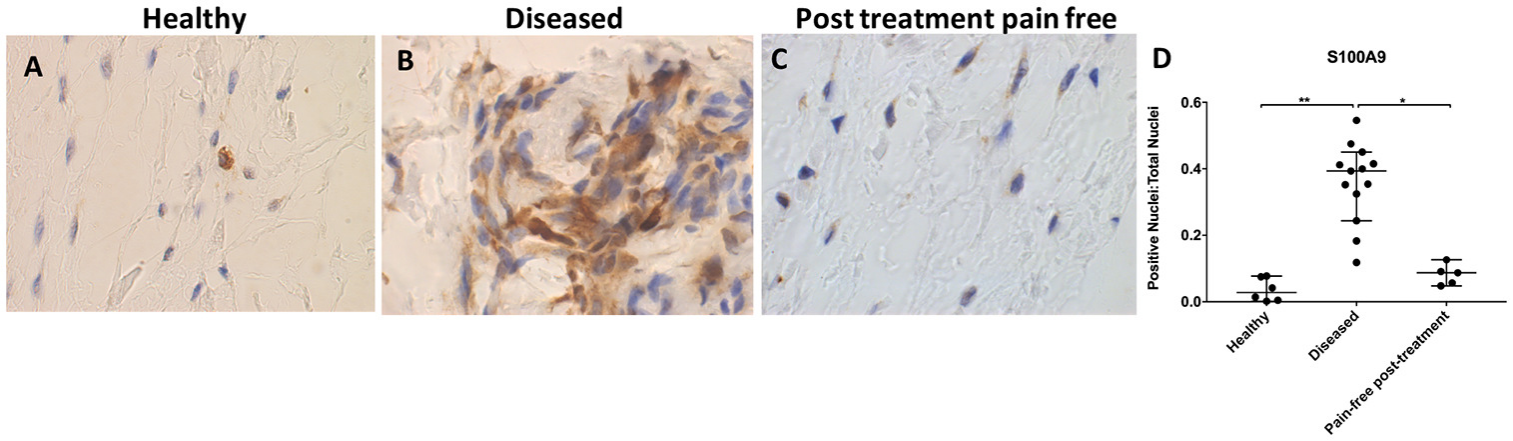
Representative images of 3,3′-diaminobenzidine immunostaining (brown) for S100A9 in (A) healthy, (B) diseased and (C) postsubacromial decompression pain-free human supraspinatus tendons. Nuclear counterstain is haematoxylin (blue). Scale bar=20 µm. (D) Quantitative analysis of immunopositive staining for S100A9. Bars represent median value with 95% CI. Data were analysed using Kruskal-Wallis test with pairwise post hoc Mann-Whitney U test; *p<0.05, **p<0.01, ***p<0.001.

S100A9 immunostaining was increased in diseased compared with healthy tendons (p=0.0002, 14.2-fold increase; figure 2A, B and D). Immunopositive staining for S100A9 showed low-level expression among tissue cells in healthy samples. S100A9 staining was reduced in tendons from post-SAD treatment patients whose pain resolved compared with diseased tissue (p=0.03, 4.5-fold decrease (figure 2C). There was no significant difference between healthy and pain-free treated tissue (p=0.97). Treated tissues showed similar characteristics to healthy samples.

### IL-33 protein expression is reduced in diseased tendons

**Figure 3:**
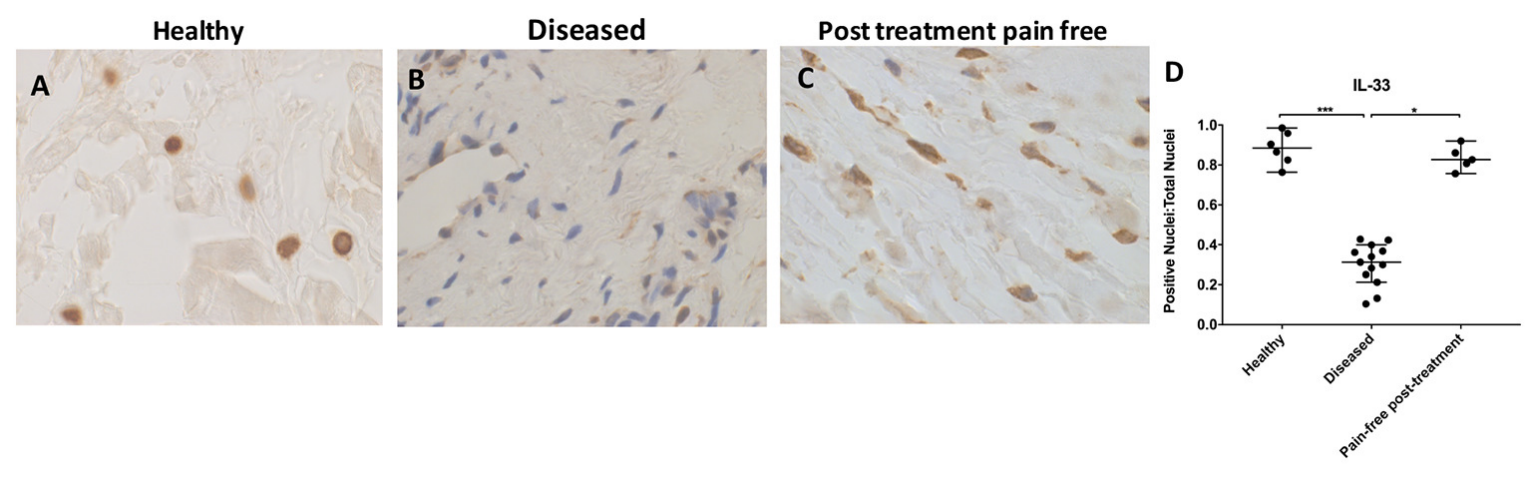
Representative images of 3,3′-diaminobenzidine immunostaining (brown) for interleukin-33 (IL-33) in (A) healthy, (B) diseased and (C) postsubacromial decompression pain-free human supraspinatus tendons. Nuclear counterstain is haematoxylin (blue). Scale bar=20 µm. (D) Quantitative analysis of immunopositive staining for IL-33. Bars represent median value with 95% CIs. Data were analysed using Kruskal-Wallis test with pairwise post hoc Mann-Whitney U test; *p<0.05, **p<0.01, ***p<0.001.

Healthy and treated tendon tissues showed similar IL-33 protein expression profiles (p>0.99; figure 3A, C and D). IL-33 staining was significantly increased in healthy compared with diseased tendons (p=0.0006, 2.8-fold increase) (figure 3B). IL-33 staining was also significantly increased in pain-free post-treatment compared with diseased tendons (p=0.01, 2.6-fold increase) (figure 2C). Immunopositive staining of IL-33 showed consistent high-level nuclear staining in healthy tendon samples. Diseased tendons showed a significant reduction in nuclear IL-33 staining compared with healthy or treated tissue; there was some cytoplasmic and extracellular staining. Treated tendon samples showed high-level nuclear IL-33 staining.

### HMGB1 protein is increased in pain-free post-treatment tendons

**Figure 4:**
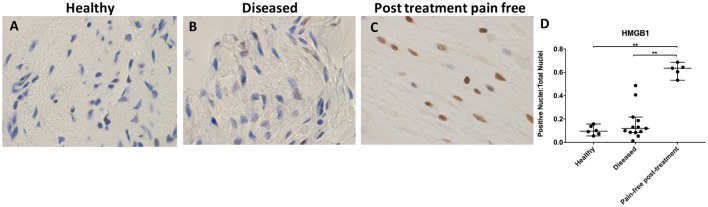
Representative images of 3,3′-diaminobenzidine immunostaining (brown) for high-mobility group box 1 (HMGB1) in (A) healthy, (B) diseased and (C) postsubacromial decompression pain-free human supraspinatus tendons. Nuclear counterstain is haematoxylin (blue). Scale bar=20 µm. (D) Quantitative analysis of immunopositive staining for HMGB1. Bars represent median value with 95% CIs. Data were analysed using Kruskal-Wallis test with pairwise post hoc Mann-Whitney U test; *p<0.05, **p<0.01, ***p<0.001.

HMGB1 expression was increased in post-treatment tendon tissues compared with healthy and diseased tendons (figure 4A-D). There was no significant difference in HMGB1 staining between healthy and diseased tendon tissues (p>0.99). Nuclear staining of HMGB1 was increased in pain-free post-treatment tissues compared with both healthy and painful diseased tissues (p=0.006 for both comparisons, 6.6-fold increase from healthy tissue, 5.3-fold increase from diseased tissue). HMGB1 immunostaining was primarily localised to the nuclear and perinuclear region in the tendon tissues studied.

### Macrophages and tendon cells express alarmins

**Figure 5:**
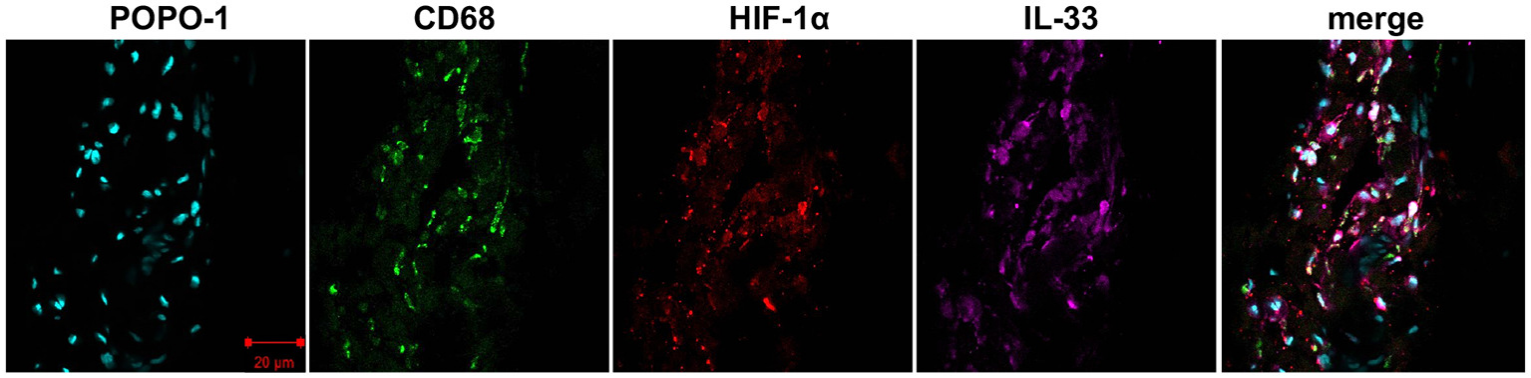
Representative confocal immunofluorescent images showing antibody labelling for macrophages (CD68, green), hypoxia-inducible factor 1α (HIF-1α) (red) and interleukin-33 (IL-33) (purple) in diseased human supraspinatus tendon tissue. Nuclear counterstain was POPO-1 (cyan). Scale bar=20 µm.

**Figure 6:**
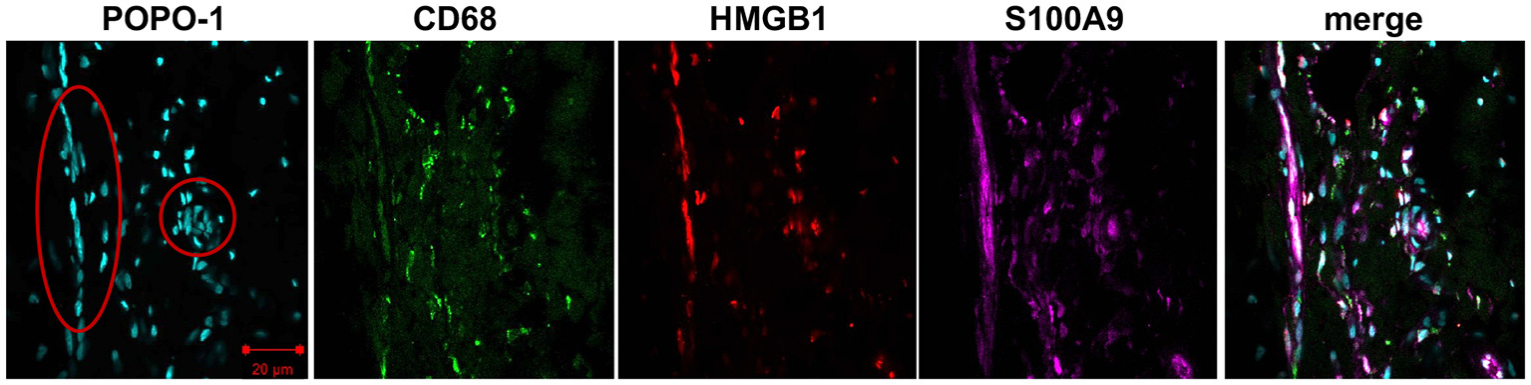
Representative confocal immunofluorescent images showing antibody labelling for macrophages (CD68, green), high-mobility group box 1 (HMGB1) (red) and S100A9 (purple) in diseased human supraspinatus tendon tissue. Nuclear counterstain was POPO-1 (cyan). Blood vessels are circled red. Scale bar=20 µm.

Given that macrophages have previously been identified in samples of diseased human supraspinatus tendons, we sought to identify if these CD68+ cells expressed alarmins and HIF-1α. Confocal images illustrating staining for HIF-1α, IL-33, S100A9, HMGB1 and CD68 are shown in figures 5 and 6. Costaining revealed that HIF-1α, S100A9 and IL-33 were expressed by CD68+ cells (macrophages) and CD68− cells, likely tendon stromal cells. HMGB1 was predominantly expressed by CD68− cells. The intensity of HMGB1 staining appeared to be more marked in perivascular regions (figure 6).

## Discussion

This study investigates protein expression in diseased tendon tissues before and after treatment and reveals pro-inflammatory roles for HIF-1α and S100A9 in tendinopathy. Additionally, IL-33 has a potentially protective role in healthy tendons that is lost with the onset of tendon disease. These findings support known mechanisms of chronic inflammation and fibrosis identified in other musculoskeletal diseases where abnormal S100A9, IL-33 and HMGB1 expression has been implicated in the initiation and propagation of various chronic inflammatory and autoimmune disorders.^18 27–29^ HMGB1 immunostaining was increased in post-treatment pain-free tendon tissues, suggesting HMGB1 may play a potential role in tissue healing.

Previous studies have illustrated that inflammation can become dysregulated over time resulting in fibrotic repair.^13 27 30 31^ There is increasing evidence that activation of the immune system after tissue injury is, in part, due to the interactions of alarmins released from necrotic cells with their associated toll-like receptors (TLRs).^32^ Alarmins have known intracellular and extracellular roles in several inflammatory signalling pathways and have been implicated in affecting resident cell phenotype.^18^ In this study, we identified S100A9, HIF-1α and IL-33 protein expression differed between healthy and diseased supraspinatus tendon tissues. Our results provide some support for the concept that dysregulation of these mediators may prime tissues to increasingly react to future inflammatory stimuli.^18 27–29 33^

HIF-1α immunostaining was significantly increased in diseased compared with healthy and treated tendon tissues. Healthy and treated tendons showed similar low-level immunostaining, although post-treatment tendons showed moderately increased levels of immunostaining than healthy tendons. These results are consistent with previous findings of HIF-1α in chronic inflammatory/autoimmune disorders and previous studies in tendon tissue.^13 20^ Stabilisation of HIF-1α with subsequent upregulation of HIF-1 is known to stimulate the release of alarmins S100A9, HMGB1 and IL-33 in inflammatory disorders.^34^ Upregulation of HIF-1 is also known to stimulate expression of vascular endothelial growth factor, which has shown to form a positive feedback loop by further stabilising HIF-1α in all cells. Extracellular HIF-1α has numerous known pro-inflammatory effects in addition to facilitating alarmin secretion: it protects myeloid cells against apoptosis and it drives macrophages towards an M1 phenotype.^35 36^ HIF-1 activation also leads to increased expression of profibrotic mediators.^28^ HIF-1α upregulation likely plays similar roles in tendon tissue: our results show that S100A9 and IL-33 dysregulation occurred alongside increased HIF-1α staining.

S100A9 staining was significantly increased in diseased tendon tissues. Previous studies have shown that extracellular S100A9 induces pro-inflammatory responses, including facilitating cell recruitment and promoting monocyte differentiation.^37 38^ S100A9 is implicated in promoting NF-κβ expression, resulting in increased secretion of tumour-necrosis factor-α (TNF-α) and interleukin-1β (IL-1β).^38 39^ Overexpression of S100A9 in ‘stressed’ tissues has been shown to result in a positive feedback mechanism resulting in downstream expression of TNF-α.^38 40^ Continued insults to tissues can induce cell phenotype changes over time and predispose tissues to failed healing: S100A9 from myeloid cells promotes further leucocyte recruitment, priming myeloid cells for more intense inflammatory responses following injuries.^38 40^ S100A9 has not previously been investigated in tendon tissues. Increased S100A9 protein in diseased tendons seen in this study is analogous to chronic inflammatory and autoimmune disorders. These findings potentially implicate S100A9 as an important initiator and propagator of chronic inflammation in tendon diseases.

IL-33 immunostaining was significantly reduced in diseased compared with healthy and treated tendons. These results are consistent with a proposed protective role of nuclear IL-33 against pro-inflammatory stimuli.^41^ Physical interaction of nuclear IL-33 with NF-κβ sequesters NF-κβ and curbs any incoming pro-inflammatory or fibrotic signals from other receptors.^41 42^ Extracellular IL-33 can stimulate interferon-γ (INF-γ) production and lead to upregulation of NF-κβ signalling pathways in infiltrating cells.^41^ However, the pro-inflammatory ‘alarmin’ role of extracellular IL-33 does not appear to be mutually exclusive with its protective nuclear function in resident cells.^41^ Our results suggest that the protective nuclear IL-33 is reduced in the resident cells of diseased tissue.

HMGB1 has become categorised as an alarmin due to its involvement in mobilisation and activation of immune cells; its overexpression has been implicated in chronic inflammatory disorders.^18 43^ Stimuli for the secretion of HMGB1 from monocytes, macrophages and dendritic cells include cellular stress, pathogen-associated molecular patterns and cytokines, including TNF-α, IL-1 and INF-γ.^17^ The primary receptors for extracellular HMGB1 are receptor for advanced glycation end products (RAGE), TLR2 and TLR4. Activation of RAGE by HMGB1 promotes chemotaxis and cytokine production through the upregulation NF-κβ pathways.^17 18 43^ HMGB1 has also been suggested to have regenerative potential. HMGB1 can induce migration of stem cells towards inflamed regions, promoting repair and regeneration.^44^

HMGB1 has not been previously investigated in diseased human tendon tissues. We hypothesised that HMGB1 staining would be increased in diseased tissues due to its pro-inflammatory and pathogenic role in some musculoskeletal disorders. Contrary to our hypothesis, HMGB1 staining was reduced in diseased tendons compared with both healthy and pain-free post-treatment tendons. These findings suggest HMGB1 may have potential role in tendon repair and regeneration, analogous to reports of HMGB1 function in diseased cardiac muscle.^44–46^

A limitation of this study was the relatively small number of patients studied in healthy and post-treatment groups. Also age-related changes may also contribute to differences in expression between healthy and diseased tendon tissues. Our healthy tendon samples were collected from younger patients and this may have influenced the findings from this study. A significant advantage of this study is the use of supraspinatus tendons in both the diseased and healthy control groups, avoiding any biological variations seen in tendons with different structure and function. Improving understanding of the role of alarmins in tendon disease will facilitate identification of potential therapeutic targets for patients with tendinopathy.

## Conclusions

In summary, we found differential protein expression of alarmins in healthy and diseased human supraspinatus tendons, before and after treatment. Specifically, S100A9 and HIF-1α may have pro-inflammatory effects in tendon disease, nuclear IL-33 may be protective against pro-inflammatory stimuli and HMGB1 may have a potential role in tendon healing. The cell types expressing S100A9, IL-33 and HIF-1α included macrophages and tendon stromal cells. Pathological activation of tendon cells through alarmin involvement may sustain chronic inflammation in tendinopathy. We propose that tendinopathy is an alarmin-mediated pathology initiated and propagated in part by increased expression of HIF-1α. Alarmin activity may facilitate recruitment of inflammatory cells in the early stages of tendon disease.
