# Supplementary material for: Differential expression of alarmins—S100A9, IL-33, HMGB1 and HIF-1α in supraspinatus tendinopathy before and after treatment
Source: BMJ Open Sport Exerc Med. 2017 May 31;3(1):e000225. doi: 10.1136/bmjsem-2017-000225 (PMC5530124; doi:10.1136/bmjsem-2017-000225)
Supplement: Supplementary table 1 [file bmjsem-2017-000225supp001.pdf]

Table S1: Primary antibodies used for immunostaining healthy, diseased and treated supraspinatus tendon tissues.

Protein	Clone	Antibody	Dilution	Species	Isotype
HIF-1α	H1alpha67	LSBio; LS-B110/26323	1:150	Mouse	IgG_{2b}
S100A9	EPR3555	ABcam; ab92507	1:200	Rabbit	IgG
IL-33		Biorbyt; orb74856	1:150	Rabbit	IgG
HMGB1	1F3	ABcam; ab190377	1:200	Mouse	IgG_{2b}
CD68	KP1	Dako; M0814	1:100	Mouse	IgG_1
